# Supplementary material for: An efficient urine peptidomics workflow identifies chemically defined dietary gluten peptides from patients with celiac disease
Source: Nat Commun. 2022 Feb 16;13:888. doi: 10.1038/s41467-022-28353-1 (PMC8850430; doi:10.1038/s41467-022-28353-1)
Supplement: Supplementary file 1 — Supplementary Information [file 41467_2022_28353_MOESM1_ESM.pdf]

Supplementary Information

An efficient urine peptidomics workflow identifies chemically defined dietary gluten peptides from patients with celiac disease

Brad A. Palanski, Nielson Weng, Lichao Zhang, Andrew J. Hilmer, Lalla A. Fall, Kavya Swaminathan, Bana Jabri, Carolina Sousa, Nielsen Q. Fernandez-Becker, Chaitan Khosla, and Joshua E. Elias

These authors contributed equally: Brad A. Palanski, Nielson Weng

These authors jointly supervised this work: Chaitan Khosla, Joshua E. Elias. Email: khosla@stanford.edu, josh.elias@czbiohub.org

This PDF file includes:

Supplementary Methods
Supplementary Figures 1 to 11
Supplementary Tables 1 to 4
Supplementary References

SUPPLEMENTARY METHODS

Method Development

Pilot Analysis of Urine from Individuals Challenged with Dietary Gluten. In LC-MS/MS-based proteomic experiments, reversed-phase extraction techniques with C18 resins are commonly used to remove salts and small molecules from peptides prior to LC-MS/MS[1]. Therefore, we initially tested a typical C18 solid phase extraction (SPE) protocol for its suitability to urine samples for gluten-derived dietary peptide analysis.

Three volunteers were recruited to eat a meal consisting of two wheat bagels and to collect spot urine samples approximately 3-4 hours thereafter. These samples underwent initial processing, storage, reduction, and alkylation as described the **Main Text Methods**. After these steps, urine (3-4.5 mL) was applied to 10 kDa cutoff centrifugal filtration units (Amicon Ultra-4, UFC800308) and centrifuged at 5000 x *g* until less than 100 µL of urine remained in the retentate. The filtrate was acidified with formic acid (FA) to a final concentration of 2% (v/v). The acidified filtrate was applied in 1 mL aliquots to C18 SPE columns (Agilent, #5982-1111) that had been pretreated with 1 mL of 100% acetonitrile and equilibrated 2 times with 1-mL aliquots of 2% aqueous FA. Bound peptides were washed with 3 x 1 mL aliquots of 2% aqueous FA. Finally, peptides were eluted in 1 mL of 60% acetonitrile/38% water/2% FA directly into microcentrifuge tubes. For all SPE steps, a vacuum manifold was used to achieve a flow rate of ~1 drop per second. Optionally, in an attempt to remove urinary pigments and metabolites, 1 mL of ethyl acetate was added to the eluent, vortexed, and then centrifuged at 16,000 x *g* to separate the aqueous and organic layers. The ethyl acetate layer was discarded. The peptide-containing aqueous layer, which had a yellow color, was flash frozen on liquid nitrogen and dried by lyophilization.

At the time of LC-MS/MS analysis, samples were reconstituted in 25 µL MilliQ water. LC-MS/MS was performed essentially as previously reported in detail[2]. Briefly, 4 µL of reconstituted peptides were separated by capillary reversed phase chromatography on a 20 cm reversed-phase column packed in-house with C18 resin on an Eksigent Ekspert nanoLC-425 or a Dionex Ultimate 3000 system using a two-step linear gradient with 4–25% buffer B (0.1% v/v FA and 5% DMSO in acetonitrile) for 20 min followed by 25–40% buffer B for 5 min. MS/MS analysis was performed using an Orbitrap Elite mass spectrometer operated in the Top 20 data-dependent acquisition mode with collision-induced dissociation for peptide fragmentation. Data were searched as described in the **Main Text Methods**, with the exception that the fragment mass tolerance was set to 0.5 Da.

Identification of a Sentinel Urine Sample for Method Optimization. For optimizing our urine peptidomic cleanup protocol, we first sought to identify a gluten-positive urine specimen as a reference sample. To do so, a 12-hour pooled urine sample was collected from a dermatitis herpetiformis patient who reported long-term (>1 month) adherence to a GFD. On the day following this urine collection, the individual repeated the 12-hour urine collection after consuming two wheat bagels (~18 g gluten). Gliadin (one of two major protein families that make up gluten) content in these urine samples was confirmed by an R5 antibody-based competitive ELISA (R7021, R-Biopharm) following the manufacturer's instructions.

Optimization of Gluten Peptide Recovery by Centrifugal Filtration. Prior reports of endogenous human urinary peptidome analysis by LC-MS/MS used centrifugal filtration to deplete high-molecular weight urinary proteins from lower-molecular weight peptides[3–6]. We first sought to verify that a similar strategy could enhance gluten peptide recovery while selecting the optimal molecular weight cutoff (MWCO). Centrifugal filters with three MWCOs were employed: 10 kDa, 30 kDa, and 50 kDa. After passing a urine sample through each filtration device, the retentate and flow-through were analyzed for gliadin peptide content by R5 ELISA, as described above, and for total protein content by SDS-PAGE and silver-stain densitometry **(Supplementary Figure 2).**

Optimization of Urinary Peptidomic Cleanup and LC-MS/MS Analysis Protocol. To maximize the number of wheat-derived peptide identifications, we systematically varied the steps in our sample preparation and LC-MS/MS analysis method (**Supplementary Figure 3a**) using 4 mL of the gluten-positive urine processed using a 10 kDa MWCO filtration device as a reference (**Supplementary Figure 2**). We endeavored to minimize carryover of urinary salts and metabolites that could lead to instrument downtime. Notably, urochrome, the major urinary pigment, has a strong yellow color[7], and we were therefore able to visually inspect samples as a proxy for successful sample cleanup, as described below.

As a starting point, reference urine was processed using Method A (**Supplementary Figure 3a**). This method is described in detail above (*Pilot Analysis of Urine from Individuals Challenged with Dietary Gluten*). After elution from the reversed-phase C18 solid phase extraction cartridge, extraction of urinary metabolites using ethyl acetate was attempted as suggested in the literature[3].

We hypothesized that some peptides remained in the retentate of the centrifugal filters due to nonspecific binding of these peptides to urinary proteins. To disrupt these putative peptide-protein interactions, 400 µL acetonitrile (ACN) was added to urine (20% final ACN concentration) just before application to the centrifugal filter. This pre-filtration denaturation step tripled the number of wheat peptide identifications. However, it resulted in the need to remove acetonitrile by drying the sample on a lyophilizer before reconstitution in 2% FA to proceed with reversed-phase C18 SPE (Method B, **Supplementary Figure 3a**). This process added one day to the overall sample workup protocol. We tested whether the addition of concentrated FA to a final concentration of 2% prior to filtration would similarly be effective to denature protein-peptide interactions (Method C, **Supplementary Figure 3a**). Method C yielded approximately the same number of wheat peptide identifications and three times the number of human peptide identifications as Method B (**Supplementary Figure 3c**), while eliminating the need for intermediate sample drying step steps. Therefore, pre-filtration acidification of urine was optimal for peptide identification. However, attempts to analyze successive urine samples prepared using Method C inevitably led to a decrease in instrument performance, presumably due to the presence of urochrome and other urinary metabolites that were not effectively removed by C18 SPE.

Recent urinary peptidomic analysis reports suggested offline HPLC fractionation by strong cation exchange (SCX) chromatography could be beneficial[5,6]. Although the rationale for this step was not explicitly stated, SCX could effectively separate urochrome, an acidic molecule, from basic peptides. Here, we sought to avoid the need for offline HPLC fractionation, as the added time would impede the throughput required for comparing large numbers of clinical samples. Instead, we sought to adapt a procedure for microscale peptide cleanup using SCX StageTips[8]. To this end, reference urine was processed by Method D, which included all steps in Method C followed by drying of the sample by lyophilization, resuspension in 100 µL 1% FA, and SCX StageTip processing (**Supplementary Figure 3a**).

To generate SCX StageTips, low-binding 200 µL pipet tips were packed with three layers of Empore SCX resin (3M, #2251) as previously described[8]. StageTips were conditioned with 100% ACN, then with 1 M NaCl, 50 mM NH_4HCO_3, and equilibrated three times with 1% FA in water. Resuspended peptide samples were applied and then washed once in 1% FA in water, then twice in 80% ACN, 18% water, and 2% FA. Peptides were eluted into low-binding microcentrifuge tubes with 500 mM NH_4COOH, 20% acetonitrile, and 0.4% FA. All steps were carried out using 100 µL of the indicated solution. Eluted peptides were dried under reduced pressure on a SpeedVac and then reconstituted in 25 µL MilliQ water. The SCX cleanup procedure appeared to effectively deplete most urochrome from the samples, as judged by a reduction in yellow color (**Supplementary Figure 3b**). Despite a slight decrease in the number of wheat peptide identifications relative to Method C (**Supplementary Figure 3c)**, we chose to include the SCX StageTip cleanup step to avoid LC-MS/MS downtime caused by urinary metabolite contamination.

Next, we optimized the LC-MS/MS acquisition method. Method D used a relatively short (25 min) LC gradient, as detailed in *Pilot Analysis of Urine from Individuals Challenged with Dietary Gluten*. An extended gradient of 90 min (Method E, **Supplementary Figure 3a**) nearly tripled the number of wheat

peptide identifications (**Supplementary Figure 3c**). For all MS analyses of urine with Methods A-E, an Orbitrap Elite instrument was used, and MS2 fragments generated by collision-induced dissociation (CID) were analyzed at low resolution in the ion trap (fragment mass tolerance used for data searches = 0.5 Da). Next, we analyzed SCX-purified urinary peptides with a similar extended LC gradient, but using an Orbitrap Fusion instrument (Method F, **Supplementary Figure 3a**). Strikingly, analysis with Method F led to a 4-fold increase in wheat peptide identifications (**Supplementary Figure 3c**). The reasons for this increase were not pursued in detail, but we speculate a primary factor is that the increased scan speed of the Orbitrap Fusion allowed us to analyze CID-generated MS2 fragment ions at high resolution in the Orbitrap (fragment mass tolerance used for data searches = 0.02 Da). While Method F improved our overall ability to identify wheat peptides approximately 20-fold compared to Method A, Method F required two days to complete due to the need to dry samples between C18 SPE and SCX StageTip processing steps. Additionally, although SCX StageTip cleanup appeared to remove the majority of urochrome (**Supplementary Figure 3b**), we still noted occasional decreases in instrument performance after attempting to analyze large batches of samples (>10).

We hypothesized that mixed cation exchange (MCX) SPE columns (Waters Corporation #186008918) that contain a mixed-mode (reversed-phase and cation exchange) resin could replace separate C18 SPE and SCX StageTip extraction steps. Accordingly, Method G (carried out as described in the **Main Text Methods)** not only appeared to completely remove urochrome from urine samples (**Supplementary Figure 3b**), but it also resulted in a ~1.5 fold increase in the number of both wheat and human peptide identifications compared to Method F. Method G also required fewer sample processing steps than Methods A-F (**Supplementary Figure 3a**), allowing us to generate LC-MS/MS ready urinary peptide samples in under 6 hours. Thus, Method G was chosen as the final analysis protocol, with a minor modification that peptides with charge state 3 and higher were selected for EThcD fragmentation in addition to CID fragmentation as detailed in the **Main Text Methods**.

SUPPLEMENTARY FIGURES

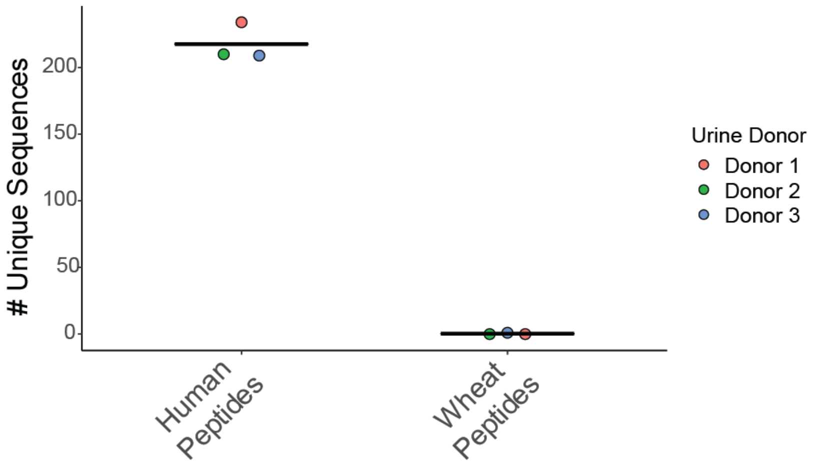

Supplementary Figure 1. A standard reversed-phase C18 SPE protocol leads to identification of few human- and wheat-derived urinary peptides. Urine samples (n=3) were processed and analyzed by LC-MS/MS as described in *Pilot Analysis of Urine from Individuals Challenged with Dietary Gluten*. Identified peptide sequences were mapped to the human and wheat proteomes as described in the **Main Text Materials and Methods**. Horizontal lines denote mean. Only a single wheat-derived peptide sequence (GQQQPFPPQQPYPQPQPFPS from α-gliadin) was detected in the urine of Donor 3. A full list of identified peptides is provided in **Supplementary Dataset 1**. Source data are provided as a Source Data file.

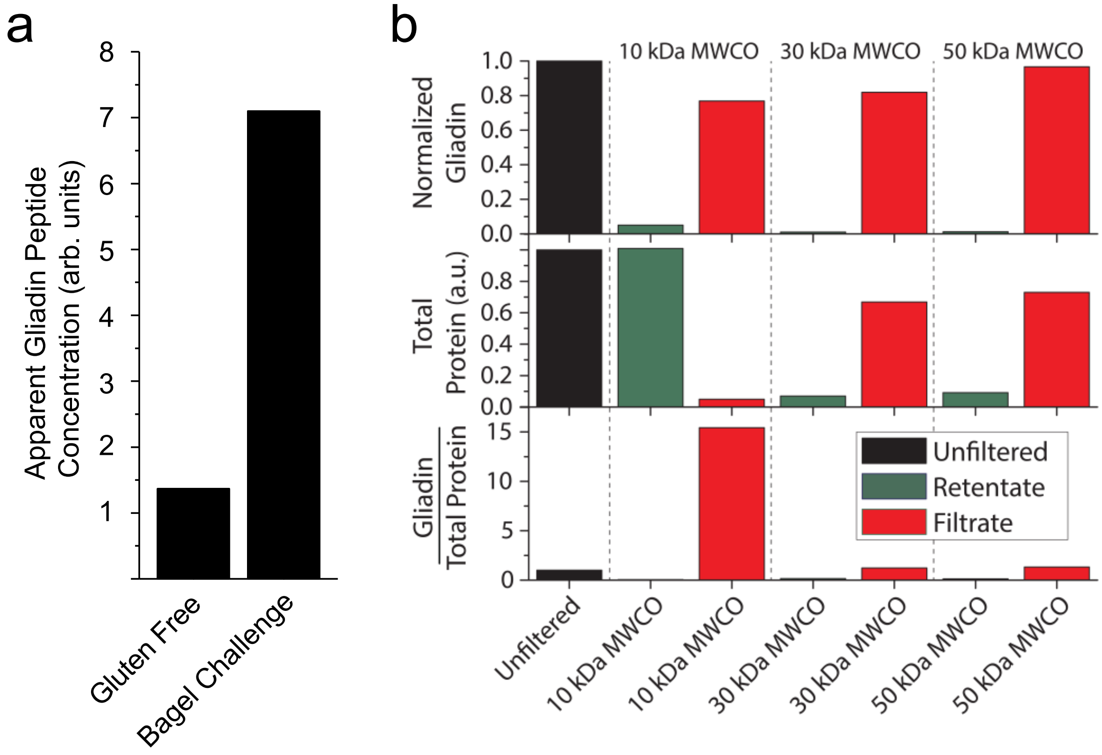

Supplementary Figure 2. Identification of a gluten-positive urine reference sample for method optimization to enhance detection of the gluten peptidome. **a** Analysis of apparent gliadin peptide content (arb. units., arbitrary units) in the urine of an individual before (Gluten Free) and after a challenge with ~18 g dietary gluten (Bagel Challenge), as measured by ELISA using the R5 antibody. An approximately 5-fold higher apparent signal in the R5 ELISA subsequent to the bagel challenge was observed relative to the gluten free sample, where the signal was presumably due to background binding of the R5 antibody to urinary proteins. This suggested that the "Bagel Challenge" sample was suitable for optimizing our sample extraction and LC-MS/MS method. **b** Urinary gliadin peptides can be recovered by ultrafiltration. The "Bagel Challenge" urine sample from **a** was passed through 10, 30, and 50 kDa centrifugal filters, and both the filtrate and retentate were analyzed. The top panel shows the gliadin content of each fraction, as evaluated by R5 ELISA, and normalized by the unfiltered urine sample. The majority of gliadin peptides pass through all ultrafiltration units. The middle panel shows the normalized protein in each fraction, as evaluated by silver-stain densiometry. For 10 kDa filtration, the majority of urinary protein is retained in the retentate. The bottom panel shows the enrichment of gliadin relative to total protein. For both the 30 and 50 kDa membranes, a majority of the R5-reactive gliadin peptides as well as the total protein content were recovered in the filtrate. In contrast, with the 10 kDa filtration device, most of the urinary protein was in the retentate, and a majority of the R5-reactive gliadin peptides passed into the filtrate, resulting in a >15-fold enrichment of gliadin peptides. Accordingly, the 10 kDa MWCO membrane appeared most suitable for efficiently recovering the gluten peptidome while depleting high molecular weight species prior to LC-MS/MS.

A

Sample Processing Workflow -->

	Pre-Filtration Denaturation	Sample Drying	Solid-Phase Extraction	EtOAc Extraction	Sample Drying	Solid-Phase Extraction	Sample Drying	LC Gradient	Orbitrap MS Instrument
**Method A**			C18 Column	✔	✔			Short	Elite
**Method B**	20% ACN	✔	C18 Column	✔	✔			Short	Elite
**Method C**	2% FA		C18 Column	✔	✔			Short	Elite
**Method D**	2% FA		C18 Column	✔	✔	SCX StageTip	✔	Short	Elite
**Method E**	2% FA		C18 Column	✔	✔	SCX StageTip	✔	Extended	Elite
**Method F**	2% FA		C18 Column	✔	✔	SCX StageTip	✔	Extended	Fusion
**Method G**	2% FA		MCX Column		✔			Extended	Fusion

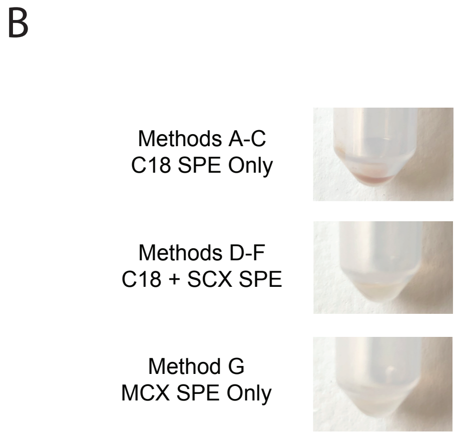

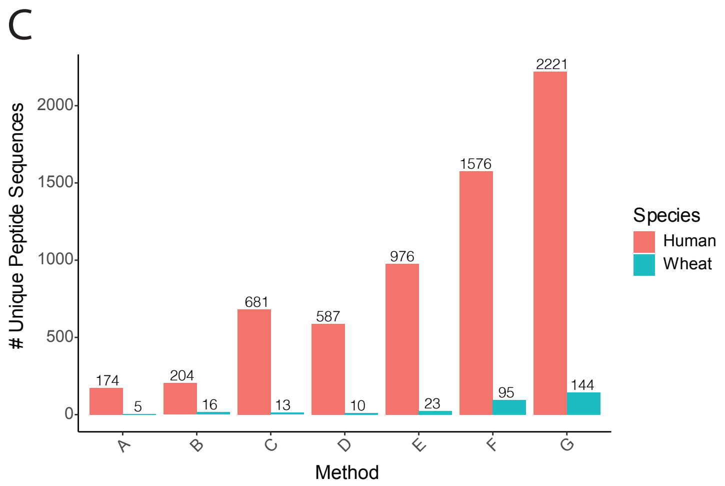

Supplementary Figure 3. Optimization of urinary peptidomic cleanup and LC-MS/MS analysis method. **a** Key steps in seven different protocols (Methods A-G) that were evaluated. The sample processing workflow moves from left to right, and a particular step was included in a method if the box is highlighted green. **b** Representative photos of urinary peptides after processing with the methods shown in **a**. Peptides processed with C18 SPE and ethyl acetate (EtOAc) extraction (Methods A-C) retained a yellow color due to carryover of urinary pigments that were not efficiently removed by SPE or liquid-liquid extraction, consistent with the low solubility of urochrome in ethyl acetate[7]. Addition of an SCX SPE step subsequent to C18 SPE (Methods D-F) appeared to remove the majority of urinary pigments. Urine extracted using a single-step MCX SPE method without ethyl acetate extraction (Method G) was completely colorless. **c** Number of human and wheat peptides identified from database searching of LC-MS/MS data after sample processing using Methods A-G. For all methods, 4 mL of the same gluten-positive reference ("Bagel Challenge") urine described in **Supplementary Figure 2** was used. A full list of the peptides identified using each Method is provided in **Supplementary Dataset 2**. Source data are provided as a Source Data file.

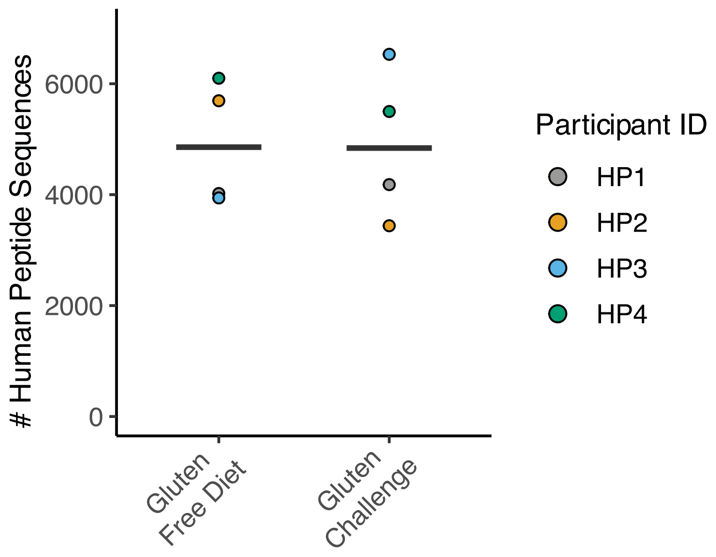

Supplementary Figure 4. Number of unique human peptide sequences identified in 8-hour pooled urine collections from 4 healthy participants (HPs) after a 24-hour gluten free diet and after challenge with dietary wheat gluten, as detailed in **Main Text Figure 2a-b**. Horizontal line indicates median. Full details related to the LC-MS/MS identification of these peptide sequences in each participants' urine sample are provided in **Supplementary Dataset 3**. Source data are provided as a Source Data file.

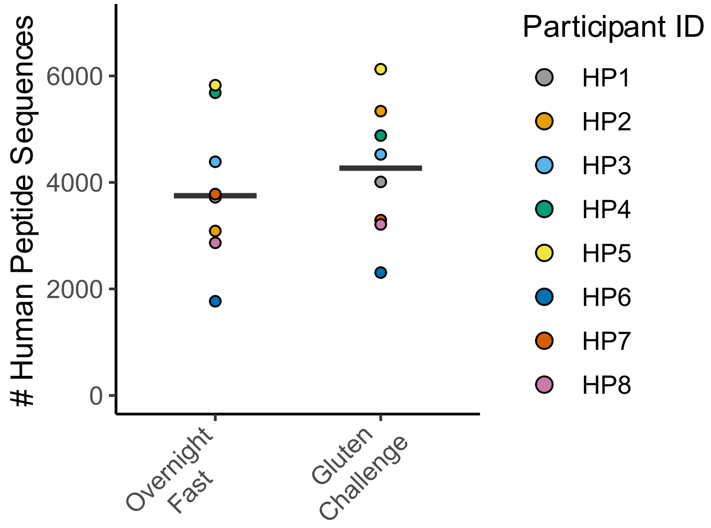

Supplementary Figure 5. Number of unique human peptide sequences identified from urine specimens collected from 8 healthy participants (HPs). Single urine specimens were collected after overnight fasting, and an 8-hour pooled urine sample was collected after a dietary gluten challenge, as detailed in **Main Text Figure 2c-d**. Horizontal line indicates median. Full details related to the LC-MS/MS identification of these peptide sequences in each participants' urine sample are provided in **Supplementary Dataset 4**. Source data are provided as a Source Data file.

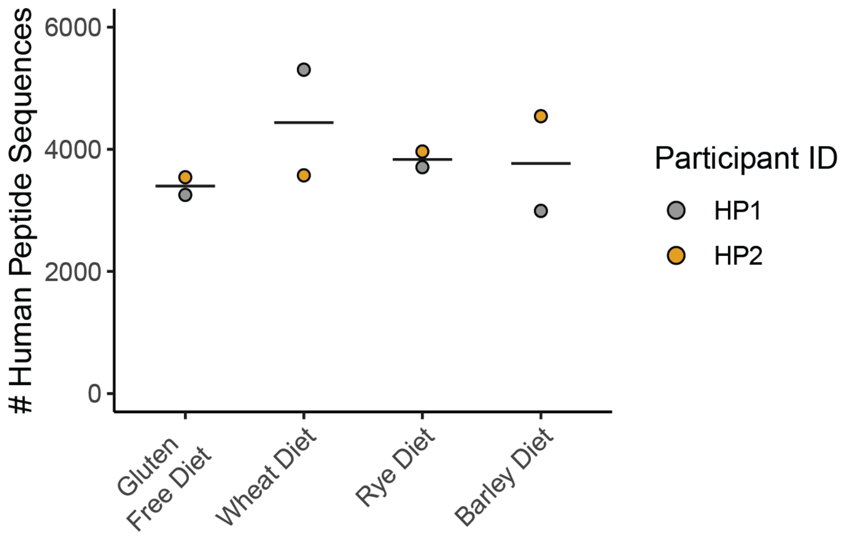

Supplementary Figure 6. Number of unique human peptide sequences identified from the urine of 2 healthy participants (HPs) in 8-hour urine collections while maintaining a gluten-free diet, or after dietary challenge with wheat, rye, or barley, as detailed in **Main Text Figure 3**. Horizontal line indicates median. Full details related to the LC-MS/MS identification of these peptide sequences in each participants' urine sample are provided in **Supplementary Dataset 5.** Source data are provided as a Source Data file.

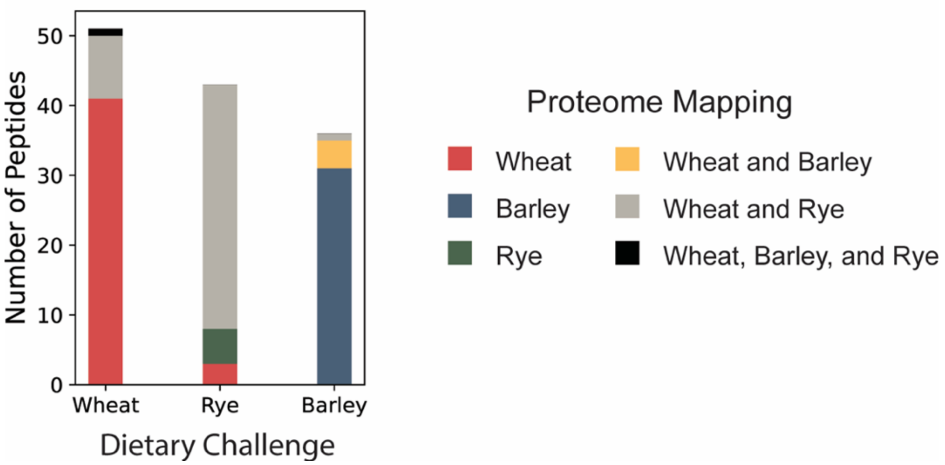

Supplementary Figure 7. Peptide-to-proteome mapping of peptides identified from LC-MS/MS analysis of urine from two healthy participants challenged with dietary wheat, rye, and barley as detailed in **Main Text Figure 3.** The large proportion of peptides uniquely mapping to the wheat proteome in the rye dietary challenege is a likely artifact of the small size of the rye proteome currently available in the Uniprot resource, which contains >100x fewer sequences than either the wheat or barley proteomes (**Main Text Methods**). A full list of observed peptide sequences in each participant and their proteome mappings is provided in **Supplementary Dataset 5.** Source data are provided as a Source Data file.

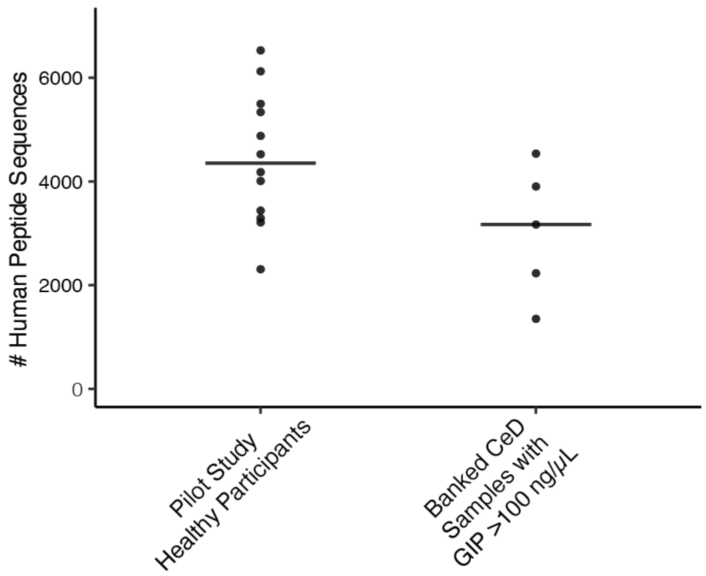

Supplementary Figure 8. Comparison of number of unique human peptide sequences identified in 8-hour urine of gluten-challenged healthy participants (pooled data from experiments in **Main Text Figure 2b,d**) and single void urine collections of banked CeD urine samples with gluten immunogenic peptide (GIP) concentrations of >100 ng/µL (data corresponds to **Main Text Figure 4**). Horizontal line indicates median. Source data are provided as a Source Data file.

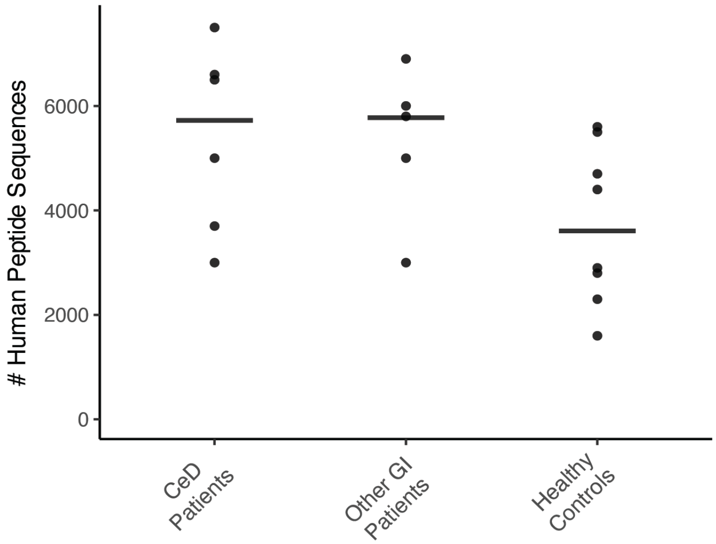

Supplementary Figure 9. Number of unique human peptide sequences identified in the urine of 6 patients with CeD, 5 patients with non-celiac gastrointestinal (GI) disorders, and 8 healthy controls in pooled urine samples collected for 8 h subsequent to a dietary challenge with two bagels. Data correspond to **Main Text Figure 5b**. Horizontal line indicates median. Differences between the three groups were not significant ($p = 0.063$, one-way Kruskal-Wallis ANOVA). Full details related to the LC-MS/MS identification of these peptide sequences in each participants' urine sample are provided in **Supplementary Dataset 7.** Source data are provided as a Source Data file.

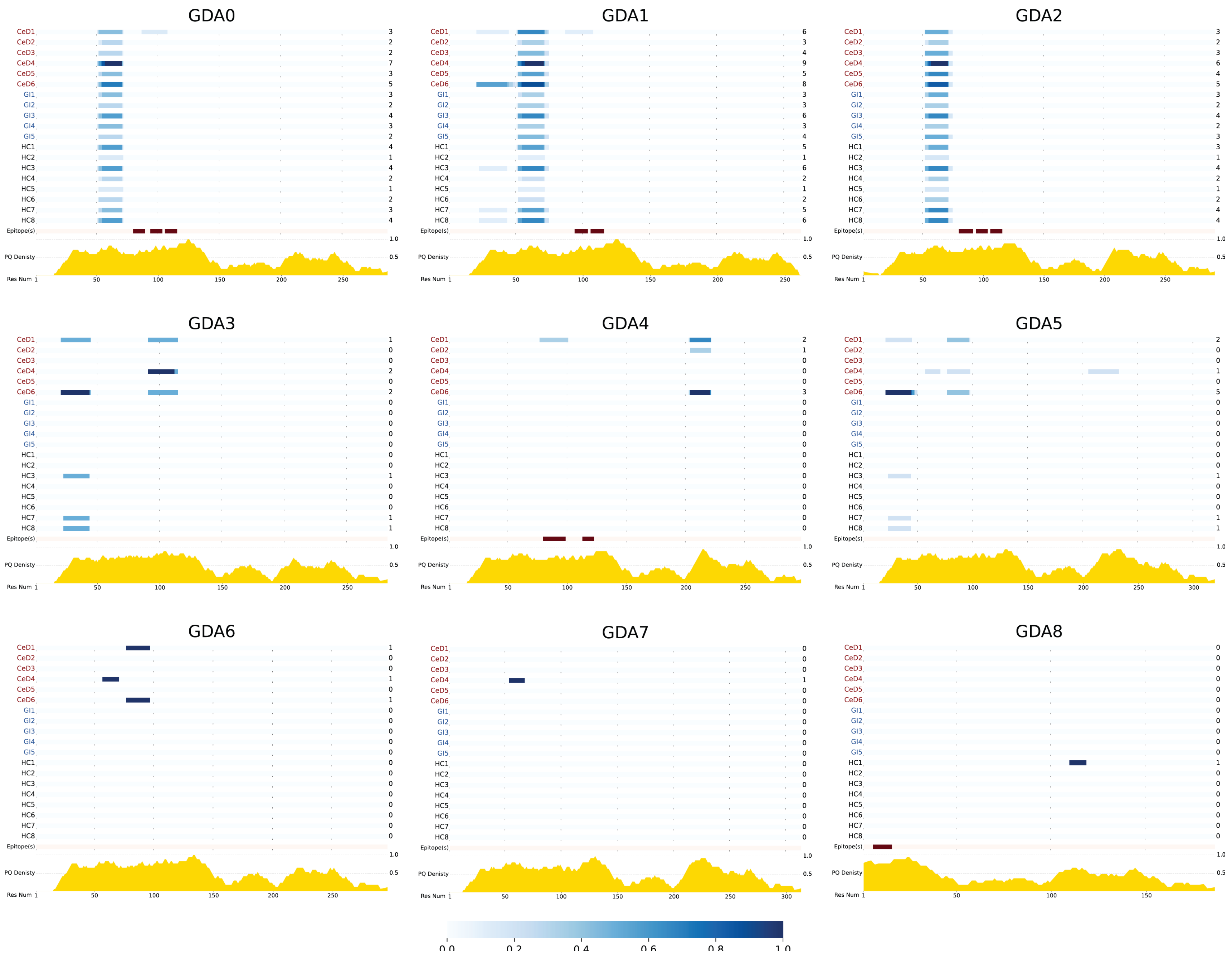
GDA0
GDA1
GDA2
GDA3
GDA4
GDA5
GDA6
GDA7
GDA8
CeD1
CeD2
CeD3
CeD4
CeD5
CeD6
GI1
GI2
GI3
GI4
GI5
HC1
HC2
HC3
HC4
HC5
HC6
HC7
HC8
Epitope(s)
PQ Denisty
Res Num
0.0
0.2
0.4
0.6
0.8
1.0

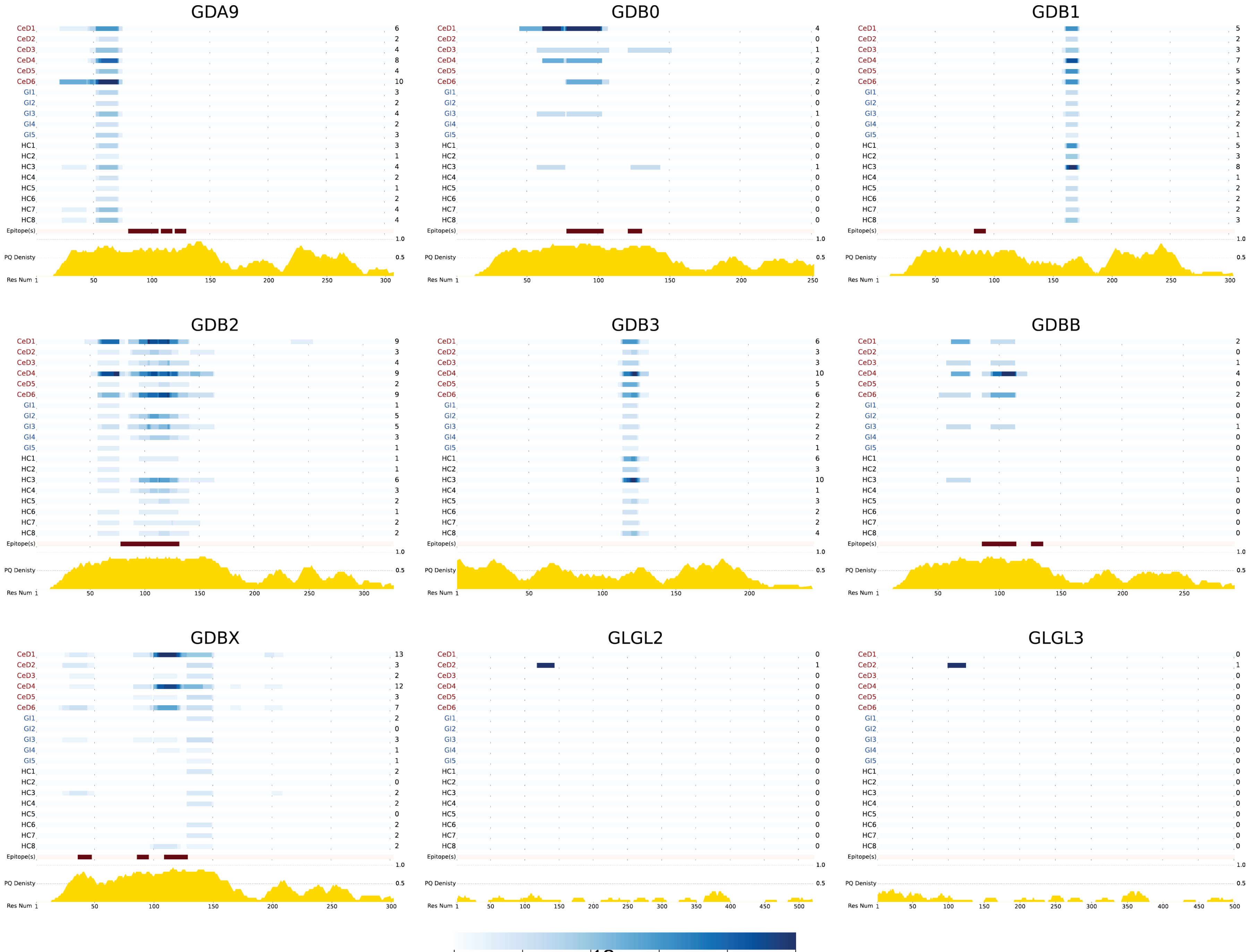

GDA9
GDB0
GDB1
GDB2
GDB3
GDBB
GDBX
GLGL2
GLGL3
CeD1
CeD2
CeD3
CeD4
CeD5
CeD6
GI1
GI2
GI3
GI4
GI5
HC1
HC2
HC3
HC4
HC5
HC6
HC7
HC8
Epitope(s)
PQ Denisty
Res Num
0.0
0.2
0.4
0.6
0.8
1.0

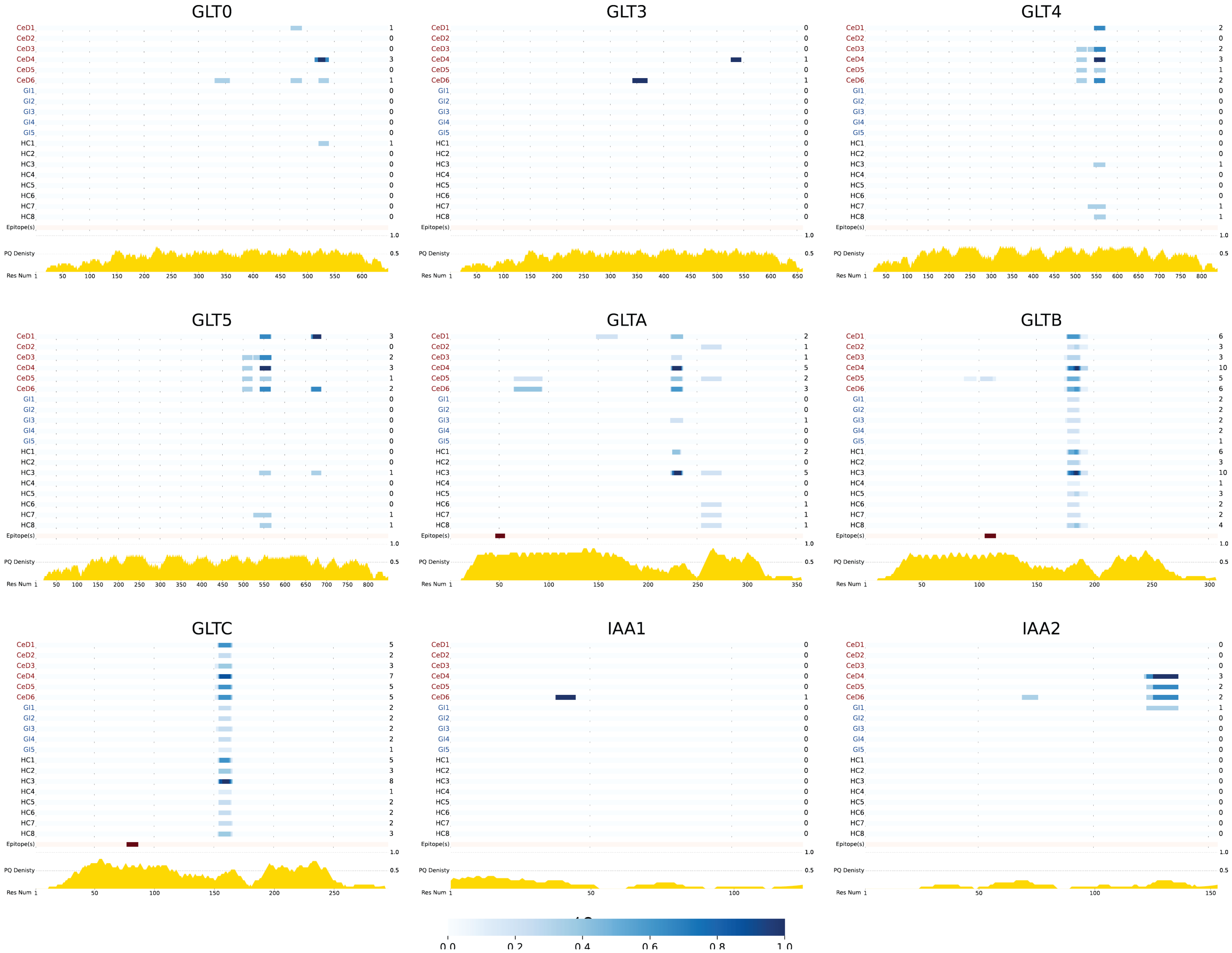
GLT0
CeD1 CeD2 CeD3 CeD4 CeD5 CeD6 GI1 GI2 GI3 GI4 GI5 HC1 HC2 HC3 HC4 HC5 HC6 HC7 HC8 Epitope(s)
1 0 0 3 0 1 0 0 0 0 0 1 0 0 0 0 0 0 0
PQ Denisty
Res Num 1 50 100 150 200 250 300 350 400 450 500 550 600
GLT3
CeD1 CeD2 CeD3 CeD4 CeD5 CeD6 GI1 GI2 GI3 GI4 GI5 HC1 HC2 HC3 HC4 HC5 HC6 HC7 HC8 Epitope(s)
0 0 0 1 0 1 0 0 0 0 0 0 0 0 0 0 0 0 0
PQ Denisty
Res Num 1 50 100 150 200 250 300 350 400 450 500 550 600 650
GLT4
CeD1 CeD2 CeD3 CeD4 CeD5 CeD6 GI1 GI2 GI3 GI4 GI5 HC1 HC2 HC3 HC4 HC5 HC6 HC7 HC8 Epitope(s)
2 0 2 3 1 2 0 0 0 0 0 0 0 1 0 0 0 1 1
PQ Denisty
Res Num 1 50 100 150 200 250 300 350 400 450 500 550 600 650 700 750 800
GLT5
CeD1 CeD2 CeD3 CeD4 CeD5 CeD6 GI1 GI2 GI3 GI4 GI5 HC1 HC2 HC3 HC4 HC5 HC6 HC7 HC8 Epitope(s)
3 0 2 3 1 2 0 0 0 0 0 0 0 1 0 0 0 1 1
PQ Denisty
Res Num 1 50 100 150 200 250 300 350 400 450 500 550 600 650 700 750 800
GLTA
CeD1 CeD2 CeD3 CeD4 CeD5 CeD6 GI1 GI2 GI3 GI4 GI5 HC1 HC2 HC3 HC4 HC5 HC6 HC7 HC8 Epitope(s)
2 1 1 5 2 3 0 0 1 0 0 2 0 5 0 0 1 1 1
PQ Denisty
Res Num 1 50 100 150 200 250 300 350
GLTB
CeD1 CeD2 CeD3 CeD4 CeD5 CeD6 GI1 GI2 GI3 GI4 GI5 HC1 HC2 HC3 HC4 HC5 HC6 HC7 HC8 Epitope(s)
6 3 3 10 5 6 2 2 2 2 1 6 3 10 1 3 2 2 4
PQ Denisty
Res Num 1 50 100 150 200 250 300
GLTC
CeD1 CeD2 CeD3 CeD4 CeD5 CeD6 GI1 GI2 GI3 GI4 GI5 HC1 HC2 HC3 HC4 HC5 HC6 HC7 HC8 Epitope(s)
5 2 3 7 5 5 2 2 2 2 1 5 3 8 1 2 2 2 3
PQ Density
Res Num 1 50 100 150 200 250
IAA1
CeD1 CeD2 CeD3 CeD4 CeD5 CeD6 GI1 GI2 GI3 GI4 GI5 HC1 HC2 HC3 HC4 HC5 HC6 HC7 HC8 Epitope(s)
0 0 0 0 0 1 0 0 0 0 0 0 0 0 0 0 0 0 0
PQ Density
Res Num 1 50 100
IAA2
CeD1 CeD2 CeD3 CeD4 CeD5 CeD6 GI1 GI2 GI3 GI4 GI5 HC1 HC2 HC3 HC4 HC5 HC6 HC7 HC8 Epitope(s)
0 0 0 3 2 2 1 0 0 0 0 0 0 0 0 0 0 0 0
PQ Density
Res Num 1 50 100 150
1.0
0.5
0.0 0.2 0.4 0.6 0.8 1.0

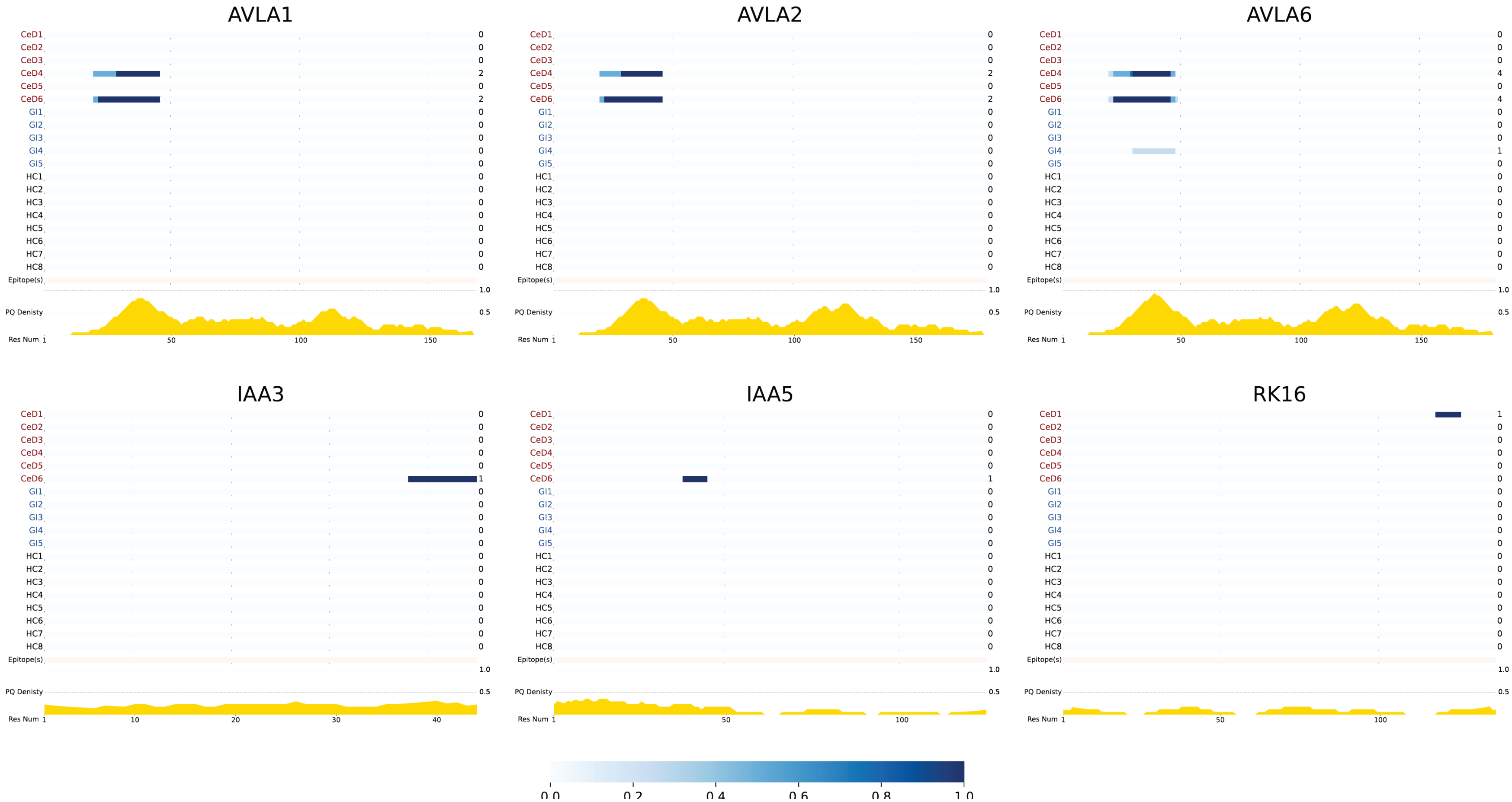

Supplementary Figure 10. Heatmap representation highlighting the locations in the wheat proteome covered by urinary peptides in each clinical study participant. Detected peptide sequences were mapped to the wheat proteome through exact sequence alignment, as shown in **Main Text Figure 5e.** For this mapping, only proteins in the wheat SwissProt proteome were considered. If a peptide's sequence aligned to multiple protein sequences or multiple sites within a single protein, then all of its potential locations in the proteome are indicated in this heatmap. Each row reflects the frequency at which the corresponding protein region was identified in each study participant's urine (CeD: patient with celiac disease; GI: patient with non-celiac gastrointestinal disorder; HC: healthy control), normalized to the maximum frequency in all participants. The frequencies shown are based on a simple count of unique peptides and are not adjusted for multiple spectrum identifications of the same peptide or intensity-based abundance inferences. Below the heatmaps, regions of the proteome that contain known CeD-relevant T-cell epitopes[9] are indicated in dark red, and the density of Pro and Gln residues (PQ density) is plotted in yellow. The density value at a given position is calculated as the fraction of residues in a window of 17 (residue at the current position, 8 proceeding residues, and 8 succeeding residues) that contains a proline or glutamine. For the first and last 8 positions of a protein sequence, preceding and succeeding window sizes are limited, respectively.

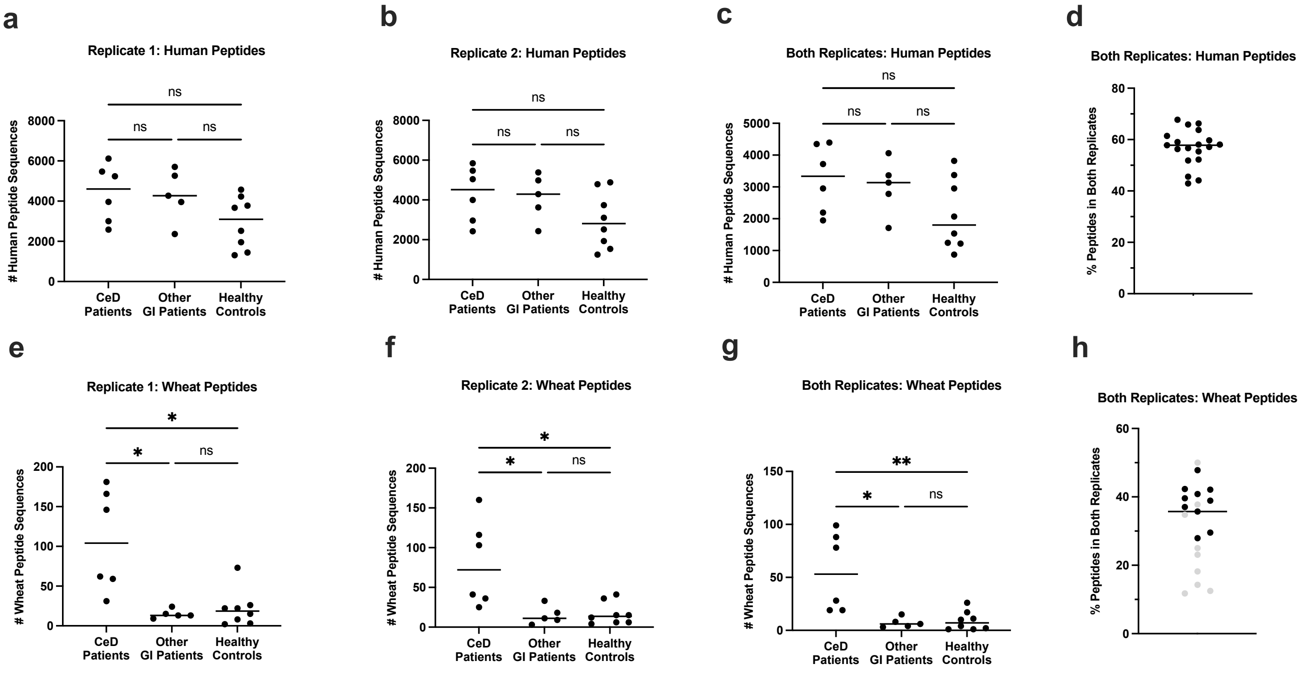

Supplementary Figure 11. Technical reproducibility of peptide identifications. Urine samples from CeD patients (n=6), patients with non-CeD gastrointestinal disorders (n=5) or healthy controls (n=8) were independently prepared on separate days and analyzed in independent LC-MS/MS runs. **a** Human peptide sequences identified in Replicate 1. **b** Human peptide sequences identified in Replicate 2. **c** Human peptide sequences identified only in both replicates. **d** Human peptide sequences identified only in both replicates expressed as a percentage of total peptides. **e** Wheat peptide sequences identified in Replicate 1. CeD patients had significantly more wheat peptides than Other GI Patients (p=0.019) and Healthy Controls (p=0.016). **f** Wheat peptide sequences identified in Replicate 2. CeD patients had significantly more wheat peptides than Other GI Patients (p=0.027) and Healthy Controls (p=0.027). **g** Wheat peptide sequences identified only in both replicates. CeD patients had significantly more wheat peptides than Other GI Patients (p=0.023) and Healthy Controls (p=0.009) **h** Wheat peptide sequences identified only in both replicates expressed as a percentage of total peptides. Samples with fewer than 20 wheat peptides identified in either replicate are denoted as gray circles. $^*p < 0.05$; $^{**}p < 0.01$, ns, not significant $p > 0.05$. One-way Kruskal-Wallis ANOVA/Dunn’s multiple comparison test. Source data are provided as a Source Data file.

SUPPLEMENTARY TABLES

Supplementary Table 1. Summary of peptides containing CeD-relevant T-cell epitopes identified in the urine of 8 healthy volunteers (from all peptide sequences listed in **Supplementary Datasets 3 and 4).**

Epitope Name(s)[a]	Epitope Sequence[a]	# Unique Peptides	Peptide Sequences with/ Epitope[b,c]
DQ2.5-glia-γ1/<br>DQ8.5-glia-γ1/<br>DQ8-glia-γ2	PQQSFPQQQ	1	PQQQFPQPQQ**PQQSFPQQQ**QPLIQPYLQQQMNPC(+57.02)KNYLLQQC(+57.02)NP
DQ2.5-glia-γ3/<br>DQ8-glia-γ1b	QQPQQPYPQ	1	FLQPQQPFP**QQPQQPYPQ**QPQQPFPQ
DQ2.5-glia-γ4b	PQPQQQFPQ	4	SQQPQQPF**PQPQQQFPQ**PQQPQ<br>SQQPQQPF**PQPQQQFPQ**PQQPQQ<br>SQQPQQPF**PQPQQQFPQ**PQQP<br>Q(-17.03)QPHQPF**PQPQQQFPQ**PQQPQQS
DQ2.5-glia-γ4c/<br>DQ8-glia-γ1a	QQPQQPFPQ	32	EQTIS**QQPQQPFPQ**QPHQPQQPYPQQQPYGSSL<br>SQQPEQTIS**QQPQQPFPQ**QPHQPQQPYPQQQPYGSSL<br>S**QQPQQPFPQ**QPHQPQQPYPQQ<br>S**QQPQQPFPQ**PQQQFPQPQQPQ<br>S**QQPQQPFPQ**PQQQFPQPQQPQQ<br>S**QQPQQPFPQ**PQQQFPQPQQP<br>S**QQPQQPFPQ**QPHQPQQPYPQQQP<br>S**QQPQQPFPQ**QPHQPQQPYPQ<br>PQLPFP**QQPQQPFPQ**PQ<br>QPQQPFPQT**QQPQQPFPQ**QPQQPFPQ<br>PQQPQLPFP**QQPQQPFPQ**PQ<br>TQQPQQPFP**QQPQQPFPQ**T<br>P[I/L]QPQQPFP**QQPQQPFPQ**P<br>P[I/L]QPQQPFP**QQPQQPFPQ**PQ<br>PLQPQQPFP**QQPQQPFPQ**QP<br>TQQPQQPFP**QQPQQPFPQ**QPQQPFP<br>AQLPFPQQP**QQPFPQPQQ**PQQPFPQ<br>S**QQPQQPFPQ**QPHQPQQPYP<br>T**QQPQQPFPQ**QPQQPFP**QQPQQPFPQ**<br>**QQPQQPFPQ**QPHQPQQPYP<br>LQQPQQPLPQP**QQPQQPFPQ**QQQPL<br>QPQQPFPQT**QQPQQPFPQ**QPQQPFP<br>T**QQPQQPFPQ**QPQQPFPQT**QQPQQPFPQ**<br>LQPQQPFP**QQPQQPFPQ**T**QQPQQPFPQ**<br>FLQPQQPFPQQPQQPYP**QQPQQPFPQ**<br>PQQPQLPFP**QQPQQPFPQ**P**QQPQQPFPQ**<br>PQQPQLPFP**QQPQQPFPQ**PQQPQQ<br>IS**QQPQQPFPQ**QPHQPQQPYPQQQPYGSSL<br>SQQLEQTIS**QQPQQPFPQ**QPHQPQQPYPQQQPYGSSL<br>PLQPQQPFP**QQPQQPFPQ**PQLPFPQQSE<br>PLQPQQPFP**QQPQQPFPQ**PQLPFPQQ<br>ESQQPFP**QQPQQPFPQ**PQ
DQ2.5-glia-γ4e	LQPQQPFPQ	7	P**LQPQQPFPQ**QPQQPFPQQP<br>**LQPQQPFPQ**QPQQPFPQTQQPQQPFPQ<br>F**LQPQQPFPQ**QPQQPYPQQPQQPFPQ<br>P**LQPQQPFPQ**QPQQPFPQPQLPFPQQSE<br>P**LQPQQPFPQ**QPQQPFPQPQLPFPQQ<br>P**[I/L]QPQQPFPQ**QPQQPFPQP<br>P**[I/L]QPQQPFPQ**QPQQPFPQPQ
DQ2.5-glia-γ5	QQPFPQQPQ	14	QPQQPFPQT**QQPQQPFPQ**QPQQPFPQ<br>T**QQPQQPFPQ**QPQQPFPQT<br>P[I/L]QPQQPFP**QQPQQPFPQ**P<br>P[I/L]QPQQPFP**QQPQQPFPQ**PQ<br>PLQPQQPFP**QQPQQPFPQ**QP<br>T**QQPQQPFPQ**QPQQPFPQQPQQPFP<br>T**QQPQQPFPQ**QPQQPFP**QQPQQPFPQ**<br>QPQQPFPQT**QQPQQPFPQ**QPQQPFP<br>T**QQPQQPFPQ**QPQQPFPQT**QQPQQPFPQ**<br>LQPQQPFP**QQPQQPFPQ**T**QQPQQPFPQ**<br>FLQPQQPFPQQPQQPYP**QQPQQPFPQ**<br>PLQPQQPFP**QQPQQPFPQ**PQLPFPQQSE<br>PLQPQQPFP**QQPQQPFPQ**PQLPFPQQ<br>ESQQPFP**QQPQQPFPQ**PQ

DQ2.5-glut-L1/<br>DQ2.2-glut-L1	PFSQQQPV	6	PQQPPFSQQQLPPFSQQLP**PFSQQQQPV**<br>PQQPPFSQQQQQQQQQQQP**PFSQQQQPV**L<br>PQQPPFSQQQQQQQQQQP**PFSQQQQPV**L<br>PQQPPFSQQQQQQQQQQP**PFSQQQQPV**<br>PQQPPFSQQQQQPILPQQP**PFSQQQQPV**<br>PQQPPFSQQQQQPILPQQP**PFSQQQQPV**L

[a]Epitope nomenclature and sequences are defined in ref. 9.
[b](+57.02) denotes cysteine alkylation by iodoacetamide during sample workup.
[c]If the UniProt database contained otherwise identical peptides containing both Leu and Ile at the same position and if the PEAKS search returned both peptides, the amino acid sequence is reported as [I/L] as these two amino acids are isobaric and indistinguishable by our MS/MS method.

Supplementary Table 2. Summary of peptides containing CeD-relevant T-cell epitopes found from the clinical study presented in **Main Text Figure 5** that were detected exclusively in patients with CeD.

**Epitope Name**[a]	**Epitope Sequence**[a]	**# Unique Peptides**	**Peptide Sequences w/ Epitope**[b]
DQ2.5-glia-α1a	PFPQPQLPY	2	Q(-17.03)LQ**PFPQPQLPY**PQPHLPYPQPQP<br>QLQ**PFPQPQLPY**PQPQLPYPQPQP
DQ2.5-glia-α1b	PYPQPQLPY	1	QLQPFPQPQL**PYPQPQLPY**PQPQP
DQ2.5-glia-α2	PQPQLPYPQ	2	Q(-17.03)LQPF**PQPQLPYPQ**PHLPYPQPQP<br>QLQPF**PQPQLPYPQ**PQLPYPQPQP
DQ2.5-glia-α3	FRPQQPYPQ	1	PYSQPQP**FRPQQPYPQ**PQPQY
DQ2.5-glia-g4a	SQPQQQFPQ	1	**SQPQQQFPQ**PQQPQQSFPQQQPPFIQPSLQQ
DQ2.5-glia-ω2	PQPQQPFPW	1	QQPQQPF**PQPQQPFPW**QPQQPFP
DQ2.5-glut-L2	FSQQQQSPF	1	Q(-17.03)ISQQQQQPPFSQQQQPP**FSQQQQSPF**SQQQQQPPFL

[a]Epitope nomenclature and sequences are defined in ref. 9.
[b](-17.03) denotes formation of pyroglutamic acid from glutamine.

Supplementary Table 3. Clinical information for patients undergoing evaluation for celiac disease corresponding to **Main Text Figure 5.**

ID	Age Group (years)	IgA Anti-TG2[a]	IgA Anti-DGP[a]	HLA Status[a]	Endoscopic Findings	Diagnosis
**GI1**	18-60	<1.0	1.2	DQ2 +/+	No villous abnormality or increased intraepithelial lymphocytosis	Non-CeD GI
**CeD1**	>60	22	>140.0	ND	Mild villous atrophy and increased intraepithelial lymphocytosis	CeD
**GI2**	18-60	<1	4	ND	No villous abnormality or increased intraepithelial lymphocytosis	Non-CeD GI
**CeD2**	18-60	52	1.4	ND	Villous blunting and increased intraepithelial lymphocytosis	CeD
**CeD3**	18-60	>128.0	73	ND	Villous blunting and increased intraepithelial lymphocytosis	CeD
**GI3**	18-60	<1.0	ND	DQ8 +/+	No villous abnormality or increased intraepithelial lymphocytosis	Non-CeD GI
**GI4**	18-60	ND	2.2	DQ2 and DQ8 -/-	Not performed due to negative HLA typing	Non-CeD GI
**CeD4**	18-60	82	ND	ND	Villous blunting and increased intraepithelial lymphocytosis	CeD
**CeD5**	18-60	47.6	54	DQ2 +/?	No villous atrophy but increased intraepithelial lymphocytosis	CeD
**CeD6**	18-60	ND	>140	ND	Severe blunting and increased intraepithelial lymphocytosis	CeD
**GI5**	>60	3.1	<1.0	DQ8 +/-	Patient refused endoscopy based on negative serology	Non-CeD GI

[a]ND, not determined.

Supplementary Table 4. Aggregated characteristics of healthy controls, patients with celiac disease (CeD), and patients with non-celiac gastrointestinal (GI) disorders from **Main Text Figure 5.**

Variable	Healthy Controls (n = 8)	Patients with CeD (n = 6)	Other GI Patients (n = 5)
**Age (%)**			
**< 18 years**	0	0	0
**18-60 years**	100	83.3	80
**>60 years**	0	16.7	20
**Age Range (years)**	22-28	20-61	20-61
**Female Sex (%)**	87.5	100	80

Supplementary Datasets 1-7 contain full information related to the identification of peptide sequences in each study participant's urine sample(s), including PEAKS software identification scores (-10lgP), detected mass-to-charge ratios (m/z), LC retention times (RT, given in minutes), and experimental mass errors (ppm compared to the theoretical m/z). Within each dataset, sheets ending in "All" contain all peptide sequences (human and wheat, and where applicable, barley and rye). Sheets ending in "Wheat" or "Grain" have those sequences that map uniquely to the wheat, and where applicable, rye and barley proteomes. These sheets containing only grain peptides have additional columns titled "Manually Corrected Peptide Sequence" and "Included in Final Dataset". These columns respectively indicate where peptide sequences were reassigned from deamidated to native or excluded entirely from analysis as described in **Validation of Wheat-Derived Peptide Sequences Identified by PEAKS Software**.

SUPPLEMENTARY REFERENCES

1. Rogers, J. C. & Bomgarden, R. D. Sample preparation for mass spectrometry-based proteomics; from proteomes to peptides. *Adv. Exp. Med. Biol.* **919**, 43-62 (2016).
2. Boucher, M. J. et al. Integrative proteomics and bioinformatic prediction enable a high-confidence apicoplast proteome in malaria parasites. *PLoS Biol* **16**, e2005895 (2018).
3. Smith, C. R. et al. Deciphering the peptidome of urine from ovarian cancer patients and healthy controls. *Clin. Proteomics* **11**, 23 (2014).
4. Sigdel, T. K. et al. Optimization for peptide sample preparation for urine peptidomics. *Clin. Proteomics* **11**, 7 (2014).
5. Di Meo, A. et al. An integrated proteomic and peptidomic assessment of the normal human urinome. *Clin. Chem. Lab. Med.* **55**, 237-247 (2017).
6. Van, J. A. D. et al. Peptidomic analysis of urine from youths with early type 1 diabetes reveals novel bioactivity of uromodulin peptides in vitro. *Mol. Cell Proteomics* **19**, 501-517 (2020).
7. Drabkin, D. L. The normal pigment of the urine. *J. Biol. Chem* **75**, 481 (1927).
8. Rappsilber, J., Mann, M. & Ishihama, Y. Protocol for micro-purification, enrichment, pre-fractionation and storage of peptides for proteomics using StageTips. *Nat. Protoc.* **2**, 1896-1906 (2007).
9. Sollid, L. M. et al. Update 2020: nomenclature and listing of celiac disease-relevant gluten epitopes recognized by $CD4^+$ T cells. *Immunogenetics* **72**, 85-88 (2020).
