# Supplementary material for: An efficient urine peptidomics workflow identifies chemically defined dietary gluten peptides from patients with celiac disease
Source: Nat Commun. 2022 Feb 16;13:888. doi: 10.1038/s41467-022-28353-1 (PMC8850430; doi:10.1038/s41467-022-28353-1)
Supplement: Supplementary file 3 — Description of Additional Supplementary Information [file 41467_2022_28353_MOESM3_ESM.pdf]

Description of Additional Supplementary Information

Title: Supplementary Dataset 1

Description: Full details related to the LC-MS/MS identification of peptide sequences in the urine of three volunteer donors (Supplementary Fig. 1), including masses and all UniProt protein accession codes.

Title: Supplementary Dataset 2

Description: Full details related to the LC-MS/MS identification of peptide sequences in the urine of an individual challenged with dietary gluten. The urine was processed with various methods shown in Supplementary Figure 3.

Title: Supplementary Dataset 3

Description: Summary of wheat peptide sequences identified in urine samples from 4 healthy participants (HPs) after a 24- hour gluten free diet (GFD) and after challenge with ~18 g dietary wheat gluten (GC), as detailed in Main Text Figure 2a-b. Full details related to the LC-MS/MS identification of these peptide sequences, including the experimentally observed masses, identification scores, and all protein accession codes, are provided.

Title: Supplementary Dataset 4

Description: Summary of wheat peptide sequences identified in urine samples from 8 healthy participants (HPs) after an overnight fast (O/N Fast) and after challenge with ~18 g dietary wheat gluten (GC), as detailed in Main Text Figure 2c-d. Full details related to the LC-MS/MS identification of these peptide sequences in each volunteer's urine sample, including the experimentally observed masses, identification scores, and all protein accession codes are provided.

Title: Supplementary Dataset 5

Description: Summary of wheat, barley, and rye peptide sequences identified in the urine of 2 healthy participants (HPs) in 8- hour urine collections while maintaining a grain-free (GF) diet, or after dietary challenge with wheat, barley, or rye, as detailed in Main Text Figure 3. Full details related to the LC-MS/MS identification of these peptide sequences in each participant's urine sample, including masses and all UniProt protein accession codes, are provided.

Title: Supplementary Dataset 6

Description: Full details related to the LC-MS/MS identification of peptide sequences identified in banked urine samples from patients with CeD (Main Text Figure 4) previously tested by lateral flow assays, including the experimentally observed masses, identification scores, and all protein accession codes, are provided.

Title: Supplementary Dataset 7

Description: Summary of wheat peptide sequences identified in the urine of 8 healthy controls (HC), 5 non-CeD GI patients, and 6 patients with CeD and after challenge with ~18 g dietary wheat gluten, as detailed in Main Text Figure 5a. Full details related to the LC-MS/MS identification of these peptide sequences, including masses and all UniProt protein accession codes, in each volunteer's urine sample are provided.

Title: Supplementary Dataset 8

Description: Wheat peptide sequences from the clinical study presented in Main Text Figure 5 that were detected exclusively in patients with CeD, ranked by the number of patients in which they appeared (n).
