# Supplementary material for: An efficient urine peptidomics workflow identifies chemically defined dietary gluten peptides from patients with celiac disease
Source: Nat Commun. 2022 Feb 16;13:888. doi: 10.1038/s41467-022-28353-1 (PMC8850430; doi:10.1038/s41467-022-28353-1)
Supplement: Supplementary file 12 — Reporting Summary (new) [file 41467_2022_28353_MOESM12_ESM.pdf]

nature portfolio

Corresponding author(s): Joshua E. Elias
Chaitan Khosla

Last updated by author(s): Jan 18, 2022

Reporting Summary

Nature Portfolio wishes to improve the reproducibility of the work that we publish. This form provides structure for consistency and transparency in reporting. For further information on Nature Portfolio policies, see our Editorial Policies and the Editorial Policy Checklist.

Statistics

For all statistical analyses, confirm that the following items are present in the figure legend, table legend, main text, or Methods section.

n/a	Confirmed	
[ ]	[x]	The exact sample size (*n*) for each experimental group/condition, given as a discrete number and unit of measurement
[x]	[ ]	A statement on whether measurements were taken from distinct samples or whether the same sample was measured repeatedly
[ ]	[x]	The statistical test(s) used AND whether they are one- or two-sided<br>*Only common tests should be described solely by name; describe more complex techniques in the Methods section.*
[x]	[ ]	A description of all covariates tested
[ ]	[x]	A description of any assumptions or corrections, such as tests of normality and adjustment for multiple comparisons
[ ]	[x]	A full description of the statistical parameters including central tendency (e.g. means) or other basic estimates (e.g. regression coefficient) AND variation (e.g. standard deviation) or associated estimates of uncertainty (e.g. confidence intervals)
[ ]	[x]	For null hypothesis testing, the test statistic (e.g. *F*, *t*, *r*) with confidence intervals, effect sizes, degrees of freedom and *P* value noted<br>*Give P values as exact values whenever suitable.*
[x]	[ ]	For Bayesian analysis, information on the choice of priors and Markov chain Monte Carlo settings
[x]	[ ]	For hierarchical and complex designs, identification of the appropriate level for tests and full reporting of outcomes
[x]	[ ]	Estimates of effect sizes (e.g. Cohen's *d*, Pearson's *r*), indicating how they were calculated

Our web collection on statistics for biologists contains articles on many of the points above.

Software and code

Policy information about availability of computer code

Data collection	No software was used in data collection.
Data analysis	Peptide-spectrum matching of LC-MS/MS data were performed using the commercial software PEAKS, versions X and X+. Selected spectra were manually validated using Thermo XCalibur, version 3.0. Downstream data processing was performed using R version 3.6.1 and Python version 3.6.8. Statistical analyses were performed using GraphPad Prism, version 9.

For manuscripts utilizing custom algorithms or software that are central to the research but not yet described in published literature, software must be made available to editors and reviewers. We strongly encourage code deposition in a community repository (e.g. GitHub). See the Nature Portfolio guidelines for submitting code & software for further information.

Data

Policy information about availability of data

All manuscripts must include a data availability statement. This statement should provide the following information, where applicable:

- Accession codes, unique identifiers, or web links for publicly available datasets
- A description of any restrictions on data availability
- For clinical datasets or third party data, please ensure that the statement adheres to our policy

The mass spectrometry data generated in this manuscript, along with the associated FASTA database files generated from the UniProt Database (http://www.uniprot.org) have been deposited to the ProteomeXchange Consortium via the PRIDE partner repository (https://www.ebi.ac.uk/pride/) with the dataset identifier PXD031048. Source data are provided with the paper.

Field-specific reporting

Please select the one below that is the best fit for your research. If you are not sure, read the appropriate sections before making your selection.

☒ Life sciences ☐ Behavioural & social sciences ☐ Ecological, evolutionary & environmental sciences

For a reference copy of the document with all sections, see nature.com/documents/nr-reporting-summary-flat.pdf

Life sciences study design

All studies must disclose on these points even when the disclosure is negative.

Sample size	No sample size calculation was performed prior to the start of the study. For our clinical study, we aimed to collect as many urine samples as possible from patients undergoing evaluation for celiac disease in the Celiac Disease Program at the Stanford Digestive Health Center in a two-year period. At the end of the recruitment period, all samples were analyzed.
Data exclusions	No data were excluded.
Replication	Samples from the clinical study presented in Main Text Fig 5. were analyzed in duplicate. Each of the duplicate urine samples was prepared on a separate day, and each sample was analyzed in a single, independent LC-MS/MS run. As shown in the full analysis of the each of the duplicate preparations (Supplementary Figure 11), both attempts at replicating our results were successful.
Randomization	Randomization is not relevant to our study. Participants were undergoing clinical evaluation for celiac disease, and urine samples were collected before a diagnosis was determined. Participants were assigned celiac disease status after full workup, in accordance with currently accepted diagnostic guidelines.
Blinding	A clinical research coordinator blinded study investigators to the disease status of the patients under evaluation for celiac disease (Main Text Figure 5) until the urine was processed and the data were analyzed.

Reporting for specific materials, systems and methods

We require information from authors about some types of materials, experimental systems and methods used in many studies. Here, indicate whether each material, system or method listed is relevant to your study. If you are not sure if a list item applies to your research, read the appropriate section before selecting a response.

Materials & experimental systems

n/a	Involved in the study	
☐	☒	Antibodies
☒	☐	Eukaryotic cell lines
☒	☐	Palaeontology and archaeology
☒	☐	Animals and other organisms
☐	☒	Human research participants
☒	☐	Clinical data
☒	☐	Dual use research of concern

Methods

n/a	Involved in the study	
☒	☐	ChIP-seq
☒	☐	Flow cytometry
☒	☐	MRI-based neuroimaging

Antibodies

Antibodies used	Gliadin (one of two major protein families that make up gluten) content in urine samples analyzed in Supplementary Figure 2 was estimated by an antibody-based competitive ELISA. The R5 antibody used in this assay was provided in the RIDASCREEN ELISA kit produced by R-Biopharm (R-7021). The antibody was used as an 11x dilution as specified by the manufacturer instructions.
Validation	Characteristics of the R5 antibody have been previously published: Osman, A. A. et al. A monoclonal antibody that recognizes a potential coeliac-toxic repetitive pentapeptide epitope in gliadins. Eur. J. Gastroenterol. Hepatol. 13, 1189-1193 (2001). Additionally, R-Biopharm, which provided the antibody in their RIDASCREEN ELISA kit has published validation data on their website (https://food.r-biopharm.com/products/ridascreen-gliadin-competitive/). In agreement with the previously published data, the manufacturer found that the antibody reacts with prolamins from wheat, rye, and barley, celiac disease related epitopes, and sequences in alpha-gliadin and gamma-gliadin.

Human research participants

Policy information about studies involving human research participants

Population characteristics	We did not analyze covariate population characteristics in this study. Information on clinical study participants is provided in Supplementary Table 3.
Recruitment	Subjects were recruited from patients undergoing evaluation for celiac disease at the Celiac Disease Program at the Stanford Digestive Health Center. The recruitment period spanned two years. The criteria for inclusion were (1) symptoms suggestive of celiac disease (e.g., dyspepsia, bloating and diarrhea) but no prior diagnosis and (2) gluten-containing diet status and willingness to undergo a defined dietary gluten challenge. The clinician and/or a clinical research coordinator asked all patients undergoing evaluation for celiac disease and meeting the inclusion criteria if they would like to participate in the study. Therefore, no self-selection or other bias is likely present. Participants were not compensated for participation.
Ethics oversight	Ethical approval for sample collections was obtained from the Institutional Review Board for Human Subject Research of Stanford University (Stanford, CA, USA) and the local ethics committee of the Hospital Universitario Virgen del Rocío (Sevilla, Spain). Participants provided informed consent.

Note that full information on the approval of the study protocol must also be provided in the manuscript.
